# Novelty detection-based approach for Alzheimer’s disease and mild cognitive impairment diagnosis from EEG

**DOI:** 10.1007/s11517-021-02427-6

**Published:** 2021-09-18

**Authors:** Matous Cejnek, Oldrich Vysata, Martin Valis, Ivo Bukovsky

**Affiliations:** 1 Department of Instrumentation and Control Engineering, Faculty of Mechanical Engineering, Czech Technical University in Prague, Technicka Street 4, 16607, Prague 6, Czech Republic; 2 Department of Neurology, Faculty of Medicine in University Hospital Hradec Králové, Charles University in Prague, Sokolská 581, 500 05 Hradec Králové, Czech Republic; 3 Department of Computer Science, Faculty of Science, University of South Bohemia in Ceske Budejovice, Ceske Budejovice, Czech Republic

**Keywords:** Novelty detection, Alzheimer’s disease, EEG, Gradient descent, Linear neural unit

## Abstract

Alzheimer’s disease is diagnosed via means of daily activity assessment. The EEG recording evaluation is a supporting tool that can assist the practitioner to recognize the illness, especially in the early stages. This paper presents a new approach for detecting Alzheimer’s disease and potentially mild cognitive impairment according to the measured EEG records. The proposed method evaluates the amount of novelty in the EEG signal as a feature for EEG record classification. The novelty is measured from the parameters of EEG signal adaptive filtration. A linear neuron with gradient descent adaptation was used as the filter in predictive settings. The extracted feature (novelty measure) is later classified to obtain Alzheimer’s disease diagnosis. The proposed approach was cross-validated on a dataset containing EEG records of 59 patients suffering from Alzheimer’s disease; seven patients with mild cognitive impairment (MCI) and 102 controls. The results of cross-validation yield 90.73% specificity and 89.51% sensitivity. The proposed method of feature extraction from EEG is completely new and can be used with any classifier for the diagnosis of Alzheimer’s disease from EEG records.

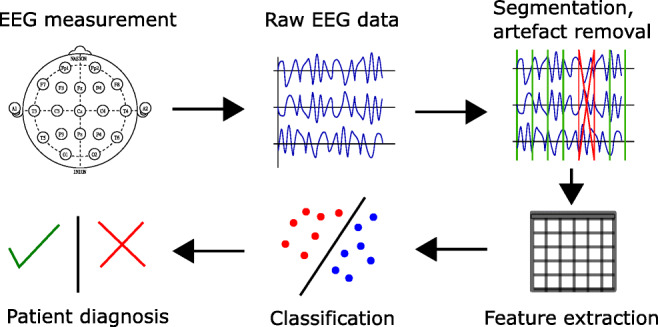

## Introduction

Alzheimer’s disease (AD) is a progressive neurodegenerative disease that is clinically characterized by impaired memory and other cognitive dysfunctions. AD is the result of brain damage that begins decades before clinical manifestations begin [1]. Currently, AD is the most common type of dementia. Previous studies have shown that this disorder is associated not only with regional brain abnormalities but also with changes in neuronal connectivity between anatomically distinct brain regions [2]. A global connectivity deficit was found in AD as it has been reported in [3]. Changes in the functional organization of the brain in patients with AD can be observed in the resting condition [4]. The hypothesis of Alzheimer’s disease as a disconnection syndrome assumes a functional or structural disconnection of larger parts of the brain rather than an isolated involvement of small areas of the brain [5]. In recent years, graph theory has been used to study anatomical and functional brain connectivity, which provides a better understanding of the relationships between different brain structures [6]. Recent studies support the hypothesis of a loss of global information integration in the AD brain due to the loss of long-distance connectivity [7]. Furthermore, the increase in theta and delta power, the decrease in beta and the slowing of the alpha frequency in AD patients were proven [8–14]. Using EEG in AD patients, it was observed a marked amplitude decrease of alpha (8–13 Hz) and an increase in power and spatial distribution in the slower delta (2–4 Hz) and theta (4–8 Hz) rhythms [15]. Association between slow-wave activity in the spectral analysis of the electroencephalogram and whole-head MEG and volumes of hippocampus in AD and MCI subjects has been observed [16, 17]. Another reported effect of AD on EEG is reduced complexity and perturbations or the decrease of EEG synchrony [14].

Mild cognitive impairment (MCI) is an intermediate stage between the expected cognitive decline of normal ageing and the more pronounced decline of dementia. It involves problems with memory, language, thinking, and judgement that are greater than typical age-related changes. However, the changes associated with MCI are not severe enough to interfere with day-to-day life and ordinary activities. Early detection of MCI may help prevent the transition to AD.

According to a previous study [18] an estimated 5.4 million Americans of all ages suffered from AD in 2016. By mid-century, the number of people living with AD in the USA is predicted to rise up to 13.8 million. Research of EEG classification for AD (and MCI) is important because it is a tool for the detection of dementia in its early stages. The early detection of dementia onset is important for establishing effective treatment [19–26]. The early diagnosis and treatment could slow down the process of dementia development [27]. For this reason, the development of easily applicable methods for diagnosis methods is still needed. EEG measurement and analysis appear to be a platform with good potential. EEG is easy to measure, non-invasive, and inexpensive.

The most common features used for the diagnosis of AD via EEG classification are frequency-domain descriptors. The review study [28] compares various classifiers using frequency-domain descriptors as it has been proposed in [29]. The results of this study (Table 1) indicate that with such features it is possible to obtain up to 94% of sensitivity and 85% specificity with known classifiers. The features obtained with deep convolutional neural networks have been used in a more recent study [30]. However, such features are impossible to interpret and computationally exhaustive to obtain. The method proposed in this study extracts the features from EEG in a computationally easy way, but it is completely different from the frequency analysis. This is the reason it can improve the classification performance of any classifier. Therefore, the goal of this study is not the classifier testing, but the analysis of the proposed feature extraction method potentials.

**Table 1:** Performance of various classifiers using frequency-domain descriptors [28]

Classifier	Sensitivity	Specificity
PC LDA	85%	85%
Stepwise PC LDA	90%	85%
PLS LDA	93%	85%
PC LR	86%	85%
PLS LR	92%	85%
Bagging	91%	85%
Random forest	91%	85%
SVM	94%	85%
Neural network	91%	85%

The proposed method is based on an adaptive novelty detection method introduced in [31] called the error and learning-based novelty detection (ELBND). The suitability of this method for non-stationary data was demonstrated in study [32]. Novelty can be considered as a measure of entropy. Different entropy measures show a decline in patients with Alzheimer’s disease [33, 34]. This study is an extension of previous conference paper [35]. In this study, we use a different dataset containing also MCI patients and data measured on two different machines — in order to determine whether this approach is independent on a device or not. A different group of AD patients was used. New patients were recorded on higher quality EEG and diagnosed in a specialized department of the University Hospital).

The ELBND method is unique. Unlike other similar methods, ELBND uses the error of a predictive model and also an increment of the adaptive weights. Only the prediction error [36, 37], or the increment of adaptive weights [38] are used in other studies. An interesting advantage of the proposed ELBND method is that even if the signal is non-stationary and of a nonlinear dynamic, the prediction model could be linear. This method was useful for the ECG signal analysis in [31]. In that report, it was proven that even the incorrectly chosen model could be sufficient for successful search for perturbations with the ELBND method.

## Materials and methods

### Participants

EEG data were obtained from 59 patients with moderate dementia (Mini-Mental State Exam (MMSE) score = 10–19) and seven patients with MCI. All patients were diagnosed according to the *National Institute of Neurological and Communicative Disorders and Stroke and the Alzheimer’s Disease and Related Disorders Association* (NINCDS-ADRDA) Alzheimer’s Criteria [39]. Clinical history was obtained from the patient and a caretaker. Information about co-morbidity at the time of diagnosis was requested from the general practitioner. A neurological and physical examination was performed in all patients. Multi-slice CT was used to assess hippocampal atrophy. Blood levels of folate, vitamin B12, thyroid stimulating hormone, calcium, glucose, complete blood cell count, renal and liver function tests were evaluated at the time of diagnosis. Serological tests for syphilis, Borrelia and HIV were made when necessary. EEG was recorded for differential diagnosis of AD to differentiate Creutzfeldt-Jakob disease or transient epileptic amnesia. CSF 14-3-3 or total tau, phospho-tau and Ab42 measurement was made in patients with rapidly progressive dementia. The Minimental State Examination Test (MMSE) was used for cognitive screening. For MCI testing the “Revised criteria for mild cognitive impairment may compromise the diagnosis of Alzheimer disease dementia” were applied [40]. Diagnosis MCI required change in cognition recognized by the affected individual or observers; objective impairment in one or more cognitive domains measured by ACE; independence in functional activities assessed by the Functional Activities Questionnaire (FAQ); and absence of dementia according to the NINCDS-ADRDA Alzheimer’s Criteria.

The control group of 102 age-matched, healthy subjects had no memory or other cognitive impairments. They failed to meet NINCDS-ADRDA Alzheimer’s criteria and showed no signs of other neurodegenerative diseases. The average MMSE of the AD group was 14.9 (standard deviation = 2.3). The mean ages of all three groups were 70.5 ± 4.9 years in the AD group, 67 ± 7.6 years in the MCI group and 72.2 ± 5.3 for the normal subjects. The structure of the groups was as follows: Alzheimer’s group, 28 men and 31 women; MCI group, 3 men and 4 women; and control group, 43 men and 59 women.

Alzheimer’s disease was diagnosed according to the “NINCDS-ADRDA Alzheimer’s Criteria” (odkaz v našem textu). Clinical history was obtained from the patient and a caretaker. Information about co-morbidity at the time of diagnosis was requested from the general practitioner. A neurological and physical examination was performed in all patients. Multi-slice CT was used to assess hippocampal atrophy. Blood levels of folate, vitamin B12, thyroid stimulating hormone, calcium, glucose, complete blood cell count, renal and liver function tests were evaluated at the time of diagnosis. Serological tests for syphilis, Borrelia and HIV were made when necessary. EEG was recorded for differential diagnosis of AD to differentiate Creutzfeldt-Jakob disease or transient epileptic amnesia. CSF 14-3-3 or total tau, phospho-tau and Ab42 measurement was made in patients with rapidly progressive dementia. The Minimental State Examination Test (MMSE) was used for cognitive screening. For MCI testing the “Revised criteria for mild cognitive impairment may compromise the diagnosis of Alzheimer disease dementia” were applied (odkaz morris). Diagnosis MCI required change in cognition recognized by the affected individual or observers; objective impairment in one or more cognitive domains measured by ACE; independence in functional activities assessed by the Functional Activities Questionnaire (FAQ); and absence of dementia according to the NINCDS-ADRDA Alzheimer’s Criteria.

The informed consent was obtained from all subjects, and the study was approved by the local ethics committee: Ethics committee Faculty Hospital Hradec Kralove. For academic projects (where no new drugs are tested and the studies are not approved by the state drug control institute) there are no assigned study numbers.

### EEG recordings and preprocessing

All recordings were performed under similar standard conditions. The subjects were in a comfortable position, on a bed, with their eyes closed. The length of the resting state recording was 15 min. Hyperventilation, photostimulation and alpha attenuation reaction were excluded from the calculation. The beginnings and ends of events such as eye opening, hyperventilation, and photostimulation in the EEG record were manually marked by the technician. During record preprocessing, sections between marks were excluded from processing. Experienced technician wakened the patients with signs of falling asleep. The electrodes were positioned according to the 10-20 System. The 10-20 system of electrode placement is a method used to describe the standardized location of scalp electrodes. It ensures that the inter-electrode spacing is equal and electrode placements are proportional to skull size and shape. The “10” and “20” refer to the 10% or 20% inter-electrode distance. Most electrode names correspond to the cerebral lobe above which they are located. The letters Fp, F, T, C, P, and O stand for Frontopolar, Frontal, Temporal, Central, Parietal and Occipital. Pre-frontal electrodes (Fp) are placed above anterior part of frontal lobe. Even numbers (2, 4, 6, 8) refer to the right hemisphere and odd numbers (1, 3, 5, 7) refer to the left hemisphere. The “z” refers to an electrode placed on the midline. The smaller the number, the closer is the position to the midline. The recording was conducted on a 21-channel digital EEG setup (Walter EEG PL-231, Germany) with a sampling frequency of 256 Hz and TruScan 32 (Alien Technik Ltd., Czech Republic) with a frequency of 128 Hz and 21-channel setup. The data in the group with recording frequency of 256 Hz were down-sampled to 128 Hz. Both groups of data were then detrended and filtered with notch filter that filters out 50 Hz. The linear detrending subtracted the best-fit line in the least-squares sense from the evaluated segments of the EEG data. The analysis began with manual artifact removal. Artifacts were rejected by experienced neurophysiologist by visual inspection. The following artifacts were eliminated by manually selecting a sample: myogenic potentials, glossokinetic artifact (important in AD patients), eye movements, ECG artifacts, pulse artifacts, respiration artifacts, skin artifacts and electrode artifacts. Afterwards, the data were grouped into non-overlapping segments of 1000 time samples (7.8125 s).

### Feature extraction

The features used for the data description were obtained from adaptive parameters of the predictive model and its error. As the predictor, we used a linear neural unit (LNU) [41, 42], with Gradient Descent (GD) adaptation [38], also known as stochastic online back-propagation.

The LNU could be described by Eq. (1):

1 $$ y(k) = \textbf{w}(k) \cdot \textbf{x}(k), $$

where *y*(*k*) is prediction output, **w**(*k*) is vector of adaptive weights (parameters) of the model and **x**(*k*) is input vector of the model. The input vector for prediction of every new sample is computed on individual EEG channels as follows:

2 $$ \textrm{\textbf{x}}(k) = [1 y_{r}(k-1) ... y_{r}(k-n) ]^{T}, $$

where *y*_*r*_(*k*) denotes measured EEG values. The input vector contains the history of last *n* time samples and bias (in this case bias = 1). The size of the used history *n* = 6 was chosen experimentally. The higher number does not produce better results but it increases the time complexity. The GD adaptation of the model Eq. (1) could be written:

3 $$ \textbf{w}(k+1)=\textbf{w}(k)+{{\varDelta}} \textbf{w}(k), $$

where *Δ***w**(*k*) is the vector of adaptive weight increments as follows:

4 $$ {{\varDelta}} \textbf{w}(k)=\mu \cdot e(k) \cdot \textbf{x}^{T}(k), $$

where the *μ* is learning rate and the error *e*(*k*) is calculated as follows

5 $$ e(k) = y_{r}(k) - y(k). $$

To improve the adaptation convergence, the measured EEG records are z-scored as follows:

6 $$ y_{r} \leftarrow Z_{3}(y_{r}) = \frac{y_{r} - \bar{y_{r}}}{3 \cdot \sigma_{yr}}, $$

where $\bar {y_{r}}$ is the mean value of *y*_*r*_, and *σ*_*yr*_ is the standard deviation of *y*_*r*_. With such a normalization, it is possible to achieve better simulation stability of weight update system [43] with a higher learning rate.

For further improvement of the adaptation convergence, we normalize the learning rate *μ* [44, 45]. For such an adaptation we used modification of the learning rate normalization as in [38] that is calculated as follows:

7 $$ \eta = \frac{\mu}{1+\mathrm{\mathbf{x}}(k)^{T} \cdot \mathrm{\mathbf{x}}(k)}, $$

where *η* is the normalized replacement for the learning rate *μ*. The learning rate adaptation is evaluated before the prediction of every individual sample. This algorithm is also called normalized least-mean-squares (NLMS).

We used the ELBND method proposed in [31], for the first time, with the normalized learning rate *η* to classify the measured EEGs. As the result of estimation, a vector of coefficients describing novelty is estimated for every sample in measured data according to the following equation:

8 $$ \mathbf{c}(k)= \left|e(k) \cdot {{\varDelta}} \mathrm{\mathbf{w}}(k)\right|= \left| e(k)^{2} \cdot \mathrm{\mathbf{x}}(k) \cdot \eta \right|. $$

If we replace *η* in Eq. (8) by Eq. (7), we will obtain

9 $$ \mathbf{c}(k)= \left| \frac{e(k)^{2} \cdot \mathrm{\mathbf{x}}(k) \cdot \mu}{\epsilon + \mathrm{\mathbf{x}}(k)^{T} \cdot \mathrm{\mathbf{x}}(k)}\right|, $$

where the regularization term *𝜖* = 1. For our work, we used just the largest coefficient out of **c**(*k*) vector to describe every sample:

10 $$ c(k) = \max(\mathbf{c}(k)). $$

The maximum was used because in some steps some weights do no increment too much. However, in general, the weights increments are strongly correlated. If we reduce the vector of increments just to its maximum we will not lose too much useful information while decreasing the dimension of the output data. The mean value estimation is alternative to maximum function; however, it does not produce better results in this case.

To make the data segments easily comparable, every segment was annotated with a single value. This further data reduction was achieved by calculating the standard deviation of *c*(*k*) coefficients for the whole EEG segment. The single value descriptor carries the important information about the novelty of the whole EEG segment. Such a simplification can be beneficial during the classification.

### Classification

The classification of positives (AD and MCI) and controls is a 2-class problem. A very simple approach was used for this classification. First, the average values of the novelty descriptor for controls and positives were calculated from the training data. The classification criteria were placed exactly between these two mean values. The subject was considered as negative or positive according to its novelty descriptor value. If the value is higher than the criteria, then the subject is classified as positive.

### Cross-validation

For the method validation, we used exhaustive leave-p-out cross-validation (*p* = 3), as it is common for a given topic. The exhaustive leave-p-out means, that we generated all possible combinations of 3 subjects from the data. For every combination, we used the chosen *p* subjects for testing and leftover subjects for training. The validation results of all combinations were subsequently used for the estimation of specificity and sensitivity.

## Results

### Novelty estimation in individual channels

First, we estimated the novelty descriptor in single EEG channels with our proposed method. The values are presented in Table 2. The values of some channels have lower standard deviations and bigger differences in mean values among the groups we wanted to classify, i.e., some channels are more suitable for the classification than others. The best channels are the ones with the biggest difference between the mean values of the normal group and the AD group or the biggest difference between the normal group and the MCI group. A solution for the problem of the best channel selection was not the goal in this study.

**Table 2:** Resulting ELBND values in normal, mild cognitive impairment (MCI) and Alzheimer disease (AD)

Ch. #	Normal	MCI	AD
Fp1	0.0297 ± 0.0059	0.0349 ± 0.0023	0.035 ± 0.0026
Fp2	0.0288 ± 0.0063	0.0372 ± 0.0061	0.0356 ± 0.0031
F7	0.0296 ± 0.0039	0.035 ± 0.0006	0.0346 ± 0.0021
Fz	0.0298 ± 0.0051	0.0349 ± 0.0029	0.0341 ± 0.0016
F3	0.0302 ± 0.005	0.0337 ± 0.0008	0.0343 ± 0.0014
F4	0.0299 ± 0.0057	0.0334 ± 0.0011	0.0342 ± 0.0016
F8	0.0294 ± 0.0045	0.0362 ± 0.0032	0.0355 ± 0.0028
T3	0.0306 ± 0.0035	0.0376 ± 0.002	0.0362 ± 0.0031
C3	0.0304 ± 0.0035	0.0347 ± 0.0013	0.0349 ± 0.002
Cz	0.0297 ± 0.0039	0.0337 ± 0.0011	0.0346 ± 0.0024
C4	0.0305 ± 0.0033	0.0352 ± 0.0019	0.035 ± 0.0017
T4	0.0306 ± 0.0035	0.0363 ± 0.0027	0.0358 ± 0.0024
T5	0.0307 ± 0.0034	0.0374 ± 0.0023	0.0364 ± 0.0033
P3	0.0302 ± 0.0027	0.0352 ± 0.0017	0.0351 ± 0.0023
Pz	0.03 ± 0.0029	0.034 ± 0.001	0.0347 ± 0.0021
P4	0.0301 ± 0.0028	0.0345 ± 0.0008	0.0352 ± 0.0018
T6	0.0299 ± 0.0026	0.0369 ± 0.0044	0.0362 ± 0.0031
O1	0.0304 ± 0.0036	0.035 ± 0.0015	0.0351 ± 0.0026
O2	0.0302 ± 0.0027	0.0351 ± 0.002	0.0352 ± 0.0026

The values are means ± standard deviations. Every row is one EEG channel. The names of the electrodes respond to the underlying lobe, part of the lobe, or position: pre-frontal (Fp), frontal (F), temporal (T), parietal (P), occipital (O), and central (C)

### Cross-validation of classification

**Fig. 1:**
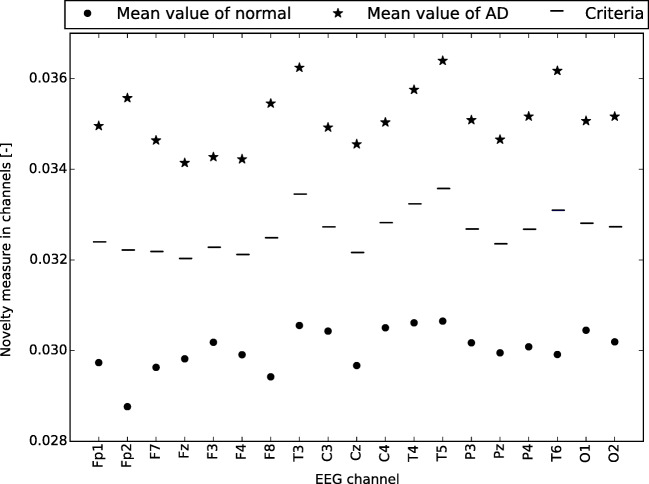
Criteria used for the classification of AD patients. The vertical axis shows the novelty measure in channels (mean value of all patients in category)

**Fig. 2:**
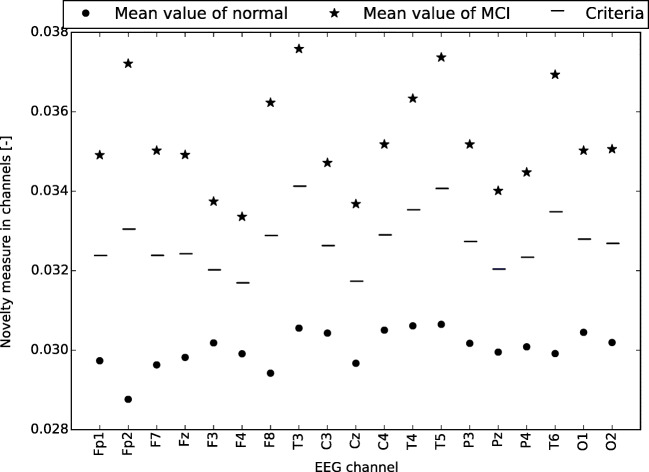
Criteria used for the classification of MCI patients. The vertical axis shows the novelty measure in channels (mean value of all patients in category)

The results of AD classification are presented in Table 3 and the MCI classification results are in Table 4. As shown, the most accurate classification was based on channels T6, P4, and P3 for AD diagnosis and was based on channels T6 and T5 for MCI diagnosis. No channel had the accuracy lower than 72% in AD classification. As far as MCI classification is concerned, all channels were above 63%. The criteria used for the classification of AD patients are shown in Fig. 1 and for MCI patients in Fig. 2.

**Table 3:** Results of the cross-validated classification of normal and AD patients according to the individual channels

Channel	Sensitivity [%]	Specificity [%]	Accuracy [%]
Fp1	91.23	67.93	76.28
Fp2	93.3	72.84	80.17
F7	92.98	76.24	82.24
F3	96.0	70.59	79.7
Fz	91.68	61.95	72.61
F4	90.83	68.63	76.59
F8	96.49	77.99	84.63
T3	85.96	80.71	82.59
C3	85.96	69.61	75.47
Cz	89.96	73.96	79.77
C4	92.98	70.75	78.72
T4	92.98	80.39	84.9
T5	85.98	84.16	84.81
P3	94.18	82.36	86.6
Pz	89.47	78.42	82.38
P4	92.98	82.35	86.16
T6	89.51	90.73	90.29
O1	84.21	83.21	83.57
O2	85.96	84.2	84.84

**Table 4:** Results of the cross-validated classification of normal and MCI patients according to the individual channels

Channel	Sensitivity [%]	Specificity [%]	Accuracy [%]
Fp1	85.71	67.76	68.91
Fp2	78.53	77.25	77.33
F7	100.0	77.47	78.92
F3	85.71	72.55	73.39
Fz	100.0	60.79	63.3
F4	85.71	67.28	68.46
F8	85.71	80.69	81.01
T3	99.43	84.33	85.3
C3	87.93	68.03	69.31
Cz	100.0	67.54	69.66
C4	100.0	71.49	73.32
T4	85.2	82.29	82.48
T5	85.71	88.32	88.15
P3	85.98	82.63	82.84
Pz	85.71	77.45	77.98
P4	100.0	78.64	80.01
T6	85.71	92.03	91.62
O1	100.0	82.69	83.8
O2	85.71	83.37	83.52

### Comparison of different machines

Because the used data were obtained from two different machines, we also compared novelty in channels to verify that the method validation results were not influenced by the source of the EEG data. The estimated values are presented in Table 5 and are estimated only for AD and MCI patients because all controls were measured with the same machine. The differences in the mean values of novelty and standard deviations of the novelty between the machines used were small or none. According to this finding it is possible to conclude that the used device does not influence the classification results.

**Table 5:** Resulting ELBND values according to the channel and EEG measuring machine (AD+MCI subjects)

Channel	Device 1 (Walter)	Device 2 (Alien)
Fp1	0.035 ± 0.003	0.035 ± 0.003
Fp2	0.036 ± 0.004	0.035 ± 0.003
F7	0.035 ± 0.002	0.034 ± 0.002
F3	0.034 ± 0.002	0.034 ± 0.001
Fz	0.034 ± 0.002	0.034 ± 0.002
F4	0.034 ± 0.002	0.034 ± 0.001
F8	0.036 ± 0.003	0.035 ± 0.003
T3	0.036 ± 0.004	0.036 ± 0.003
C3	0.035 ± 0.002	0.035 ± 0.002
Cz	0.034 ± 0.003	0.034 ± 0.002
C4	0.035 ± 0.002	0.035 ± 0.002
T4	0.036 ± 0.003	0.036 ± 0.002
T5	0.037 ± 0.004	0.036 ± 0.003
P3	0.035 ± 0.003	0.035 ± 0.002
Pz	0.035 ± 0.002	0.034 ± 0.002
P4	0.035 ± 0.002	0.035 ± 0.002
T6	0.037 ± 0.004	0.035 ± 0.002
O1	0.035 ± 0.003	0.035 ± 0.002
O2	0.035 ± 0.003	0.035 ± 0.002

## Discussion

From the clinical point of view, the most important is timely and inexpensive diagnosis of individuals at risk of developing AD. MCI is associated with a high risk of developing AD. Therefore, the authors retain this group of patients separately.

The reason for the lower age of patients with MCI is the age dependence of AD, where MCI in many cases results in AD. The lower age of MCI patients could reduce the sensitivity of the test in this group. In the case of comparable age average of MCI with other groups, we would expect an increase in the value of the discriminatory test.

The most significant changes were found in the brain areas with the most expressed neurohistological changes (temporal regions). Episodic memory is the function most commonly impaired early in AD as a consequence of mesial temporal lobe atrophy (entorhinal cortex, hippocampus) which disables consolidation. MRI features involve two features: mesial temporal lobe atrophy (particularly the hippocampus, entorhinal cortex and perirhinal cortex) and temporoparietal cortical atrophy [46]. So the first and most affected parts of the brain in the typical Alzheimer’s disease are temporoparietal regions. The neurohistological changes (such as amyloid plaques and neurofibrillary tangles, neuropil threads, and dystrophic neurites) cannot be verified by computed tomography data. The authors of this study proceeded from the typical localization of these changes, which is the basis of the diagnosis of Alzheimer’s disease [47]. The hypothesis that both groups — the positives (AD+MCI) and the controls — have the same spectral power in Delta and Theta bands was tested with t-test. Welch’s method was used for the power spectral analysis of a signal. The resulting p-values were tested with false discovery rate (FDR) to control the false discoveries due to multiple comparisons problem. P-values for all the channels were under the FDR threshold of 0.05. This might indicate the brain atrophy in the AD group.

The previously displayed results [35] obtained with different datasets yield a better classification rate (sensitivity and specificity of 95%). This may be caused by multiple reasons: bigger and more balanced dataset in the previous study, origin of all data from one measuring device, absence of MCI patients, classification over all channels. The goal of this study was to analyze the novelty in separate channels and evaluate the influence of measuring device on the classification performance and to estimate the potential of this method.

The truism that EEG is nonspecific and cannot diagnose aetiology or localization well is often cited. However, in general medical practice, non-specificity is often not the question because most of the referrals in general neurology are individuals in whom the cause is clear, or reasonably suspected, on the basis of clinical history and laboratory chemistry. The questions from the clinician are whether the brain is involved and what the extent of the brain damage is. The novelty estimation may bring new information about the changes in the brain dynamics during cognitive decline in patients with AD. It may become a suitable complement to the traditional qEEG methods.

## Conclusion

The desynchronization of the EEG is the interruption of its rhythmical activity. It occurs with the activation of ascending cholinergic projections of the basal forebrain and brainstem and projections from the raphe nuclei and locus coeruleus [48–50]. The rhythmical activity is interrupted both by direct effects on cortical neurons and by indirect effects on thalamic neurons. The cholinergic hypothesis, which was initially presented 20 years ago, suggests that a dysfunction of acetylcholine containing neurons in the brain might substantially contribute to the cognitive decline observed in patients with Alzheimer’s disease. Thus, the decreased cholinergic projections to the cerebral cortex in patients with cognitive decline make the desynchronization of the EEG activity less probable. Unlike the EEG energies, where the maximum changes are present in most of the atrophic parts of the brain, the novelty changes are more diffuse and probably reflect the effects of diffuse cholinergic projections on the cortical oscillatory activity. The distribution of changes was similar to those changes in complex noise characteristics [51].

The proposed method was able to mark measured EEGs with a single value that could be directly used for the AD diagnosis. The current work points to less complexity at smaller scales in AD group in frontal areas, while higher complexity at larger scales was observed across the brain areas and this higher complexity was significantly correlated with cognitive decline [52]. It is well known that EEG signals of AD patients are generally less synchronous than in age-matched control subjects [53]. Lower predictability (higher level of novelty) may reflect a higher complexity of EEG signal in patients with AD.

The presented results show that our proposed method has the accuracy comparable with other methods using different EEG features (Table 1). However, a sample of seven MCI patients is very small to extract any meaningful result. The results, however, give hope that this methodology could be sensitive to MCI. To test this hypothesis it is still necessary to expand the group of patients with MCI. In this paper, we also compare the values produced by the proposed method on the data obtained from two different machines.

The data from one of the machines was also resampled. According to the results of comparison, it appears that results are independent of the measuring device and resampling process.

## References

[1] Dubois B, Hampel H, Feldman HH, Scheltens P, Aisen P, Andrieu S, Bakardjian H, Benali H, Bertram L, Blennow K (2016). Preclinical alzheimer’s disease: definition, natural history, and diagnostic criteria. Alzheimers Dement.

[2] He Y, Chen Z, Gong G, Evans A (2009). Neuronal networks in alzheimers disease. Neuroscientist.

[3] Morrison JH, Scherr S, Lewis DA, Campbell M, Bloom FE, Rogers J, Benoit R (1986) The laminar and regional distribution of neocortical somatostatin and neuritic plaques: implications for alzheimer’s disease as a global neocortical disconnection syndrome. Biol Substrates Alzheimers Dis :115–131

[4] Sorg C, Riedl V, Mühlau M, Calhoun VD, Eichele T, Läer L, Drzezga A, Förstl H, Kurz A, Zimmer C (2007). Selective changes of resting-state networks in individuals at risk for alzheimer’s disease. Proc Natl Acad Sci.

[5] Palesi F, Castellazzi G, Casiraghi L, Sinforiani E, Vitali P, Gandini Wheeler-Kingshott CA, D’Angelo E (2016). Exploring patterns of alteration in alzheimer’s disease brain networks: a combined structural and functional connectomics analysis. Front Neurosci.

[6] Stam CJ, Reijneveld JC (2007). Graph theoretical analysis of complex networks in the brain. Nonlinear Biomed Phys.

[7] Sanz-Arigita EJ, Schoonheim MM, Damoiseaux JS, Rombouts SA, Maris E, Barkhof F, Scheltens P, Stam CJ (2010). Loss of ’small-world’networks in alzheimer’s disease: graph analysis of fmri resting-state functional connectivity. PloS ONE.

[8] Adler G, Brassen S, Jajcevic A (2003). Eeg coherence in alzheimer’s dementia. J Neural Transm.

[9] Jelic V, Johansson S-E, Almkvist O, Shigeta M, Julin P, Nordberg A, Winblad B, Wahlund L-O (2000). Quantitative electroencephalography in mild cognitive impairment: longitudinal changes and possible prediction of alzheimer’s disease. Neurobiol Aging.

[10] Claus J, Kwa V, Teunisse S, Gérard J, Van Gool W, Hans J, Koelman T, Bour L, De Ongerboer Visser B (1998). Slowing on quantitative spectral eeg is a marker for rate of subsequent cognitive and functional decline in early alzheimer disease. Alzheimer Dis Assoc Disord.

[11] Coben L, Chi D, Snyder A, Storandt M (1990). Replication of a study of frequency analysis of the resting awake eeg in mild probabke alzheimer’s disease. Electroencephalogr Clin Neurophysiol.

[12] Duffy F, Albert M, McAnulty G (1984). Brain electrical activity in patients with presenile and senile dementia of the alzheimer type. Ann Neurol.

[13] Ihl R, Dierks T, Martin E-M, Frölich L, Maurer K (1996). Topography of the maximum of the amplitude of eeg frequency bands in dementia of the alzheimer type. Biol Psychiatry.

[14] Dauwels J, Vialatte F, Cichocki A (2010). Diagnosis of alzheimer’s disease from eeg signals: where are we standing?. Curr Alzheimer Res.

[15] Klimesch W (1999). Eeg alpha and theta oscillations reflect cognitive and memory performance: a review and analysis. Brain Res Rev.

[16] Fernández A, Arrazola J, Maestú F, Amo C, Gil-Gregorio P, Wienbruch C, Ortiz T (2003). Correlations of hippocampal atrophy and focal low-frequency magnetic activity in alzheimer disease: volumetric mr imaging-magnetoencephalographic study. Am J Neuroradiol.

[17] Helkala E-L, Hänninen T, Hallikainen M, Könönen M, Laakso M, Hartikainen P, Soininen H, Partanen J, Partanen K, Vainio P (1996). Slow-wave activity in the spectral analysis of the electroencephalogram and volumes of hippocampus in subgroups of alzheimer’s disease patients. Behav Neurosci.

[18] Association A (2016). 2016 alzheimer’s disease facts and figures. Alzheimers Dement.

[19] Staudinger T, Polikar R (2011) Analysis of complexity based eeg features for the diagnosis of alzheimer’s disease. In: Engineering in medicine and biology society, EMBC, 2011 Annual international conference of the IEEE. IEEE, pp 2033–2036 10.1109/IEMBS.2011.6090374 22254735

[20] Stevens A, Kircher T (1998). Cognitive decline unlike normal aging is associated with alterations of eeg temporo-spatial characteristics. Eur Arch Psychiatry Clin Neurosci.

[21] Elgendi M, Vialatte F, Cichocki A, Latchoumane C, Jeong J, Dauwels J (2011) Optimization of eeg frequency bands for improved diagnosis of alzheimer disease. In: Engineering in medicine and biology society, EMBC, 2011 annual international conference of the IEEE. IEEE, pp 6087–6091 10.1109/IEMBS.2011.6091504 22255728

[22] Strik WK, Chiaramonti R, Muscas GC, Paganini M, Mueller TJ, Fallgatter AJ, Versari A, Zappoli R (1997). Decreased eeg microstate duration and anteriorisation of the brain electrical fields in mild and moderate dementia of the alzheimer type. Psychiatry Res Neuroimaging.

[23] Müller T, Thome J, Chiaramonti R, Dierks T, Maurer K, Fallgatter A, Frölich L, Scheubeck M, Strik W (1997). A comparison of geeg and hmpao-spect in relation to the clinical severity of alzheimer’s disease. Eur Arch Psychiatry Clin Neurosci.

[24] Akrofi K, Baker MC, O’Boyle MW, Schiffer RB (2008) Clustering and modeling of eeg coherence features of alzheimer’s and mild cognitive impairment patients. In: Engineering in medicine and biology society, 2008. EMBS 2008. 30th Annual international conference of the IEEE. IEEE, pp 1092–1095 10.1109/IEMBS.2008.4649350 19162853

[25] de Waal H, Stam CJ, de Haan W, van Straaten EC, Scheltens P, van der Flier WM (2012). Young alzheimer patients show distinct regional changes of oscillatory brain dynamics. Neurobiol Aging.

[26] Iznak A, Kolykhalov I, Zhygulskaya S, Vasilieva A, Selezneva A, Selezneva N (1998). The quantitative eeg in early and differential diagnosis of mild dementia of different genesis. Eur Neuropsychopharmacol.

[27] Henderson G, Ifeachor E, Hudson N, Goh C, Outram N, Wimalaratna S, Del Percio C, Vecchio F (2006). Development and assessment of methods for detecting dementia using the human electroencephalogram. IEEE Trans Biomed Eng.

[28] Lehmann C, Koenig T, Jelic V, Prichep L, John RE, Wahlund L-O, Dodge Y, Dierks T (2007). Application and comparison of classification algorithms for recognition of alzheimer’s disease in electrical brain activity (eeg). J Neurosci Methods.

[29] Herrmann W, Fichte K, Freund G (1979). Reflections on the topics: Eeg frequency bands and regulation of vigilance. Pharmacopsychiatry.

[30] Morabito FC, Campolo M, Ieracitano C, Ebadi JM, Bonanno L, Bramanti A, Desalvo S, Mammone N, Bramanti P (2016) Deep convolutional neural networks for classification of mild cognitive impaired and alzheimer’s disease patients from scalp eeg recordings. In: 2016 IEEE 2nd International Forum on Research and technologies for society and industry leveraging a better tomorrow (RTSI). IEEE, pp 1–6

[31] Cejnek M, Beneš PM, Bukovsky I (2014) Another adaptive approach to novelty detection in time series

[32] Cejnek M, Bukovsky I (2018). Concept drift robust adaptive novelty detection for data streams. Neurocomputing.

[33] Cao Y, Cai L, Wang J, Wang R, Yu H, Cao Y, Liu J (2015). Characterization of complexity in the electroencephalograph activity of Alzheimer’s disease based on fuzzy entropy. Chaos Interdiscip J Nonlinear Sci.

[34] Deng B, Liang L, Li S, Wang R, Yu H, Wang J, Wei X (2015). Complexity extraction of electroencephalograms in alzheimer’s disease with weighted-permutation entropy. Chaos Interdiscip J Nonlinear Sci.

[35] Cejnek M, Bukovsky I, Vysata O (2015) Adaptive classification of eeg for dementia diagnosis. In: 2015 International workshop on IEEE computational intelligence for multimedia Understanding (IWCIM), pp 1–5

[36] Bishop CM (1994) Novelty detection and neural network validation. In: IEE Proceedings vision, image and signal processing, vol 141. IET, pp 217–222

[37] Williams G, Baxter R, He H, Hawkins S, Gu L (2002) A comparative study of rnn for outlier detection in data mining. In: Null. IEEE, p 709

[38] Bukovsky I, Oswald C, Cejnek M, Benes PM (2014) Learning entropy for novelty detection a cognitive approach for adaptive filters. In: Sensor signal processing for defence (SSPD) 2014, pp 1–5

[39] McKhann GM, Knopman DS, Chertkow H, Hyman BT, Jack CR, Kawas CH, Klunk WE, Koroshetz WJ, Manly JJ, Mayeux R (2011). The diagnosis of dementia due to alzheimer’s disease: Recommendations from the national institute on aging-alzheimer’s association workgroups on diagnostic guidelines for alzheimer’s disease. Alzheimers Dement.

[40] Morris JC (2012). Revised criteria for mild cognitive impairment may compromise the diagnosis of alzheimer disease dementia. Arch Neurol.

[41] Gupta M (2003). Static and dynamic neural networks: from fundamentals to advanced theory.

[42] Gupta M, Bukovsky I, Homma N, Solo AMG, Hou Z-G (2013) Fundamentals of higher order neural networks for modeling and simulation. In: Fundamentals of higher order neural networks for modeling and simulation. IGI Global, pp 103–133

[43] Bukovskỳ I, Rodriguez R, Bila J, Homma N (2012) Prospects of gradient methods for nonlinear control, Automatizácia a riadenie v teórii a praxi ARTEP 2012

[44] Widrow B (1985). Adaptive signal processing, ser. Prentice-Hall signal processing series.

[45] Mandic DP, Goh VSL (2009). Complex valued nonlinear adaptive filters: Noncircularity, Widely linear and neural models.

[46] Patel KP, Wymer DT, Bhatia VK, Duara R, Rajadhyaksha CD (2020). Multimodality imaging of dementia: Clinical importance and role of integrated anatomic and molecular imaging. RadioGraphics.

[47] Hyman BT, Trojanowski JQ (1997). Editorial on consensus recommendations for the postmortem diagnosis of alzheimer disease from the national institute on aging and the reagan institute working group on diagnostic criteria for the neuropathological assessment of alzheimer disease. J Neuropathol Exp Neurol.

[48] Zaborszky L, Pang K, Somogyi J, Nadasdy Z, Kallo I (1999). The basal forebrain corticopetal system revisited. Ann N Y Acad Sci.

[49] Fuller P, Sherman D, Pedersen NP, Saper CB, Lu J (2011). Reassessment of the structural basis of the ascending arousal system. J Comp Neurol.

[50] Berntson G, Shafi R, Sarter M (2002). Specific contributions of the basal forebrain corticopetal cholinergic system to electroencephalographic activity and sleep/waking behaviour. Eur J Neurosc.

[51] Vyšata O, Procházka A, Mareš J, Rusina R, Pazdera L, Vališ M, Kukal J (2014). Change in the characteristics of eeg color noise in alzheimer’s disease. Clin EEG Neurosci.

[52] Mizuno T, Takahashi T, Cho RY, Kikuchi M, Murata T, Takahashi K, Wada Y (2010). Assessment of eeg dynamical complexity in alzheimer’s disease using multiscale entropy. Clin Neurophysiol.

[53] Dauwels J, Vialatte F, Musha T, Cichocki A (2010). A comparative study of synchrony measures for the early diagnosis of alzheimer’s disease based on eeg. Neuroimage.

